# Healthy Eating Design Guidelines for School Architecture

**DOI:** 10.5888/pcd10.120084

**Published:** 2013-02-28

**Authors:** Terry T-K Huang, Dina Sorensen, Steven Davis, Leah Frerichs, Jeri Brittin, Joseph Celentano, Kelly Callahan, Matthew J. Trowbridge

**Affiliations:** Author Affiliations: Terry T-K Huang, Leah Frerichs, Jeri Brittin, University of Nebraska Medical Center College of Public Health, Omaha, Nebraska; Dina Sorensen, Steven Davis, Joseph Celentano, Kelly Callahan, VMDO Architects, Charlottesville, Virginia.

## Abstract

We developed a new tool, Healthy Eating Design Guidelines for School Architecture, to provide practitioners in architecture and public health with a practical set of spatially organized and theory-based strategies for making school environments more conducive to learning about and practicing healthy eating by optimizing physical resources and learning spaces. The design guidelines, developed through multidisciplinary collaboration, cover 10 domains of the school food environment (eg, cafeteria, kitchen, garden) and 5 core healthy eating design principles. A school redesign project in Dillwyn, Virginia, used the tool to improve the schools’ ability to adopt a healthy nutrition curriculum and promote healthy eating. The new tool, now in a pilot version, is expected to evolve as its components are tested and evaluated through public health and design research.

## Introduction

Creating school food environments that facilitate healthy eating among children is a recommended national strategy to prevent and reduce childhood obesity (1). According to socioecological models, the macroenvironment — the density of fast food outlets around schools (2,3), for example — affects eating behavior. Limited research has focused on the microenvironment, such as building design. School design can affect student behavior, development, and academic performance (4–6). Food displays and time allotment for school meals can also affect children’s eating behavior (7–9). A recent evaluation of a system-level healthy eating initiative in 4 California schools showed that changes in dining room design and features may have contributed to positive outcomes such as increased nutrition and knowledge of the food environment, preference for fruits and vegetables, and higher in-school and out-of-school fruit and vegetable consumption (10).

Interest is growing in how the physical design of school buildings (ie, architecture, interior design, and landscaping) affects school policies and practices and the subsequent eating behaviors and norms among children. Systematic theory- and evidence-based design strategies drawn from both public health and architecture are needed to define, test, and further develop best practices. We developed a pilot version of a new tool, Healthy Eating Design Guidelines for School Architecture. These guidelines, organized for practical use by architects, schools, and health researchers, present a set of design strategies to help promote healthy eating behaviors in schools.

## Development of the Healthy Eating Design Guidelines for School Architecture

### Theoretical frameworks

The Healthy Eating Design Guidelines (Table) represent a new application of existing theoretical frameworks on the role of school building design in child development and health promotion (11). The guidelines draw on research in environmental health, environmental psychology, behavioral economics, and socioecological models.

**Table:** Spatial Domains, Design Strategies, and Core Healthy Eating Design Principles, Healthy Eating Design Guidelines for School Architecture, Pilot Version, 2013

**Domain 1: Commercial Kitchen Zone**	
**Objective: **Design an open commercial kitchen to facilitate the procurement, preparation, and storage of fresh, organic, whole foods that are prepared in a manner to preserve nutritional value.	
**Design strategy**	**Core Principle^a^**
Articulate the kitchen area as a demonstration kitchen with an open view to food preparation stations from servery and seating zones.	4
Create dedicated display and storage areas for fresh and preserved fruits and vegetables.	4
Design freezer and refrigeration capacity to accommodate seasonally available, locally sourced food, including food from federally subsidized school programs such as Farm-to-Schools.	1
Provide kitchen equipment such as ovens, tilt skillets, and steamers that allows for a variety of cooking methods for fresh foods.	1
Avoid deep-fat fryers.	1
Provide kitchen equipment that allows for a variety of processing and preservation methods, such as canning and freezing of fresh foods.	1
Provide storage bins for a variety of whole grains and whole grain flours.	1
Provide flash-freezing capacity for fresh local foods.	1
Provide sufficient counter or work space for processing of fresh foods.	1
**Domain 2: Teaching Kitchen Zone**	
**Objective:** Design complementary hands-on teaching kitchen areas for students and extracurricular organization use.	
**Design strategy**	**Core Principle^a^**
Create a visual and/or physical connection to the commercial teaching kitchen, seating area, and outdoor school gardens.	4
Provide areas conducive to teaching, presentation, and demonstration cooking.	2
Create teaching kitchen as a hands-on learning environment with equipment that is safe and accessible to children.	2
Create an outdoor kitchen area conducive to traditional (historical) and experimental teaching and cooking (ie, open-fire cooking, solar oven).	2
Provide outdoor kitchen with access to potable water.	2
**Domain 3: Serving Zones**	
**Objective: **Design the servery to function efficiently to maximize dining time for students, while effectively encouraging the selection and enjoyment of healthy foods and beverages.	
**Design strategy**	**Core Principle^a^**
Provide servery space for healthy grab-and-go meal options in the snack or express line.	1
Provide space behind the servery counter for packaged snacks to be served on request only.	1
Use mobile hot and cold servery equipment carts for flexibility and a variety of arrangements (eg, freestanding fresh salad and fruit station in seating areas).	1
Avoid servery equipment that serves exclusively competitive foods (eg, self-serve ice cream freezers).	1
Provide age-appropriate self-service food preparation stations (eg, juicing, microwaving, toasting).	3
Place healthy foods at eye level of children, and specify food service equipment that allows one to do so.	3
Include servery lines in sufficient number to ensure efficient user flow, thereby ensuring all students have adequate time to eat. Coordinate with district wellness policy.	3
Provide visual circulation cues to support efficient flow through servery areas.	3
Situate disposal areas to avoid conflicts with users entering the servery or dining areas. Arrange disposal areas along dining area exit route, when possible.	3
Provide express checkout lanes for students choosing healthy meals, with no sugary or salty products such as sweetened beverages, chips, or desserts.	3
Position servery equipment to accommodate nutritious foods (eg, broccoli) at the beginning of the server line.	3
Design space by cafeteria register to allow for display of healthy foods and minimize child access to foods high in fat and sugar.	3
Provide servery equipment that can accommodate changeable food descriptors/labels.	3
Provide servery equipment that provides space for multiple healthy choices in each food category(celery *and* carrots).	3
Provide servery equipment with closed sides and tops when sale of less healthful options is required. (ie, ice cream).	3
Position salad bars away from walls for 360-degree circulation.	3
Position salad bars near the checkout register.	3
Provide servery counter space that can accommodate fruit bowls for serving fresh fruits and vegetables.	3
Provide space for serving trays.	3
**Domain 4: Dining Zones**	
**Objective: **Reconceive dining areas as places of enjoyment and relaxation, conceived in such a way as to fully support healthy food initiatives.	
**Design strategy**	**Core Principle^a^**
Create visual access between dining areas and other food spaces (eg, school garden and/or commercial kitchen).	4
Create a variety of seating options and social arrangements, recognizing that not all students will be comfortable in a given configuration.	3
Provide outdoor seating areas designed for the local climate (ie, covered or shaded, as necessary) and connected to the interior dining area.	3
Design dining areas to recognized national standard for seating capacity, to avoid overcrowding.	3
Provide comfortable seating.	3
Provide small refrigerators in every classroom, for storage of packed snacks, lunches, and beverages.	1
Provide staff refrigerators in proximity to anticipated staff eating areas.	1
**Domain 5: Aesthetics of Healthy Food Environments**	
**Objective: **Design spaces to provide a relaxing atmosphere conducive to the enjoyment of food and social interaction.	
**Design strategy**	**Core Principle^a^**
Feature fresh, preserved, or prepared food in public spaces.	4
Incorporate appealing colors and lighting.	3
Provide targeted acoustic treatments with high noise reduction coefficients in public gathering spaces such as dining areas.	3
Incorporate integrated audio capabilities that allow music to be played in selected areas.	3
**Domain 6: Educational Signage, Way Finding, and Marketing**	
**Objective: **Deploy graphic design and signage elements throughout the school environment to reinforce the healthy eating message.	
**Design strategy**	**Core Principle^a^**
Incorporate visible and educational indicators of school (or municipal) water quality.	4
Design architectural interiors to provide dedicated space for healthy nutrition marketing (eg, corridors, stairways, servery, dining areas).	4
Provide daily/weekly/monthly menu signage at the entry to the dining area and servery zone and throughout the seating zone.	4
Provide educational (nutritional) information on food choices. Highlight information on seasonal fresh foods incorporated into the school food program.	4
Locate educational (nutritional) signage so that it is visible from the “point of choice” in the servery zone.	4
Prescreen nutritional marketing to eliminate potentially competitive foods (eg, chocolate “Got Milk?” posters).	4
**Domain 7: Water Access and Vending Machines**	
**Objective: **Support healthy eating using design and policy strategies focused on the school physical environment that facilitate access to drinking water and discourage unhealthy food and drink choices from vending machines.	
**Design strategy**	**Core Principle^a^**
Place vending machines selling unhealthy options away from dining and primary traffic areas (visually and spatially).	3
Provide ready access to potable water and cups in dining areas.	1
Place drinking fountains in outdoor activity areas.	1
Place drinking fountains near social/public areas.	1
Provide potable water in every classroom.	1
Incorporate advanced filtration system for the school’s potable water supply.	1
Provide free potable water sources at a rate of 1 per 100 occupants.	1
Provide at least 50% water sources conducive to filling water bottles.	1
Provide storage space for refillable water containers.	1
Replace vending machine content with healthy food and beverage options.	1
**Domain 8: On-Site Food Production**	
**Objective: **Provide spaces for on-site food cultivation and production, coordinated with curricular and extracurricular activities.	
**Design strategy**	**Core Principle^a^**
Create a school garden.	2
Create a school farming facility (producing, for example, tilapia, honey, or eggs).	2
Create a greenhouse facility for educational purposes and/or support of the school garden.	2
Use edible plantings for landscaping.	2
Include on-site food production resources (eg, garden, greenhouse) in construction documents for building facility, where possible.	2
**Domain 9: Integrated Healthy Food Education Facilities**	
**Objective: **Identify and provide programming opportunities to extend healthy food messaging throughout the school.	
**Design strategy**	**Core Principle^a^**
Provide a school wellness center readily accessible to all students, designed to support nutritional counseling, and integrated with related school functions such as the health educator or school nurse.	4
Design science laboratories conducive to food-related experiments (eg, soils laboratory).	4
Maintain a library collection dedicated to healthy eating and nutrition.	4
Design food spaces to support curricular, extracurricular, and community education.	4
Provide dedicated space for educational materials in clear view of all students.	4
Incorporate Internet access or kiosk for nutritional information and research.	4
**Domain 10: Integrated Healthy Food Community**	
**Objective: **Support healthy eating and local food production in the community.	
**Design strategy**	**Core Principle^a^**
Design food spaces for flexibility and multiple uses by school, school affiliates, and community groups.	5
Provide community garden space for local use.	5
Provide mobile/modular units that enable rapid reconfiguration of the dining area.	5
Host community farmers’ market on school grounds.	5

a Core principles: 1) provide equipment and spaces that facilitate the incorporation of fresh and healthy food choices into the school and its community; 2) provide facilities to directly engage the school community in food production and preparation; 3) apply evidence- and theory-based behavioral science principles to “nudge” the school community toward healthy eating behaviors and attitudes; 4) use building and landscape features to promote awareness of healthy and sustainable food practices; 5) conceive and articulate school spaces as community assets to multiply the benefits of school-based healthy food initiatives.

The history of integrating environmental health research into architectural practice is well-documented. For example, factors such as air quality, acoustics, climate, crowding, ergonomics, and lighting are routine considerations for their direct effects on occupant well-being. Environmental factors can affect activity patterns, stress, appetite, and food choices. Environmental psychology recognizes the transactional relationship between the built environment and social life (12). For example, the shift toward open-space planning within schools in the 1970s necessitated a divergence from traditional teaching styles (5,13,14). The field of proxemics explores how physical space influences social interactions (15). Occupants of space take an active role in constructing the meanings of that space, which, in turn, may influence behaviors and culture (13). Changes to school classroom features, such as the introduction of reading nooks, affect both teaching methods and student learning behaviors (4). Behavioral economics investigates environmental cues, such as the organization of food service delivery and point-of-purchase prompts, which may implicitly motivate decisions on healthy food purchase and intake (16,17). Socioecological models consider these concepts beyond the classroom. Schools play a vital and visible role in their communities; the physical design of a school and its grounds may affect interactions among different ecological levels, including parents, community members, and community policies.

### Collaborative process

The Healthy Eating Design Guidelines were developed through iterative collaboration between the disciplines of public health and architecture. Development began in early 2010 to inform the joint redesign of Buckingham Primary and Buckingham Elementary Schools in Dillwyn, Buckingham County, Virginia, by VMDO Architects. The project was commissioned to target cutting-edge environmental sustainability goals while providing an optimized healthy food environment for students, staff, and the community.

**Figure 1:**
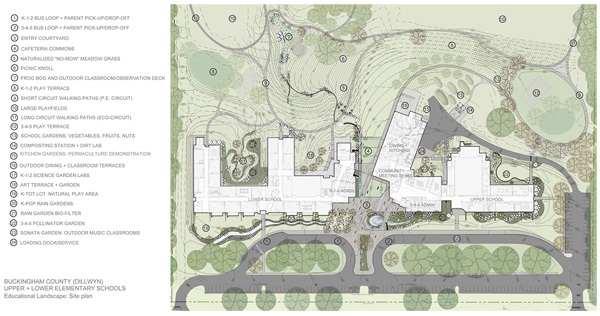
Site plan for upper and lower elementary schools in Dillwyn, Virginia.

**Figure 2:**
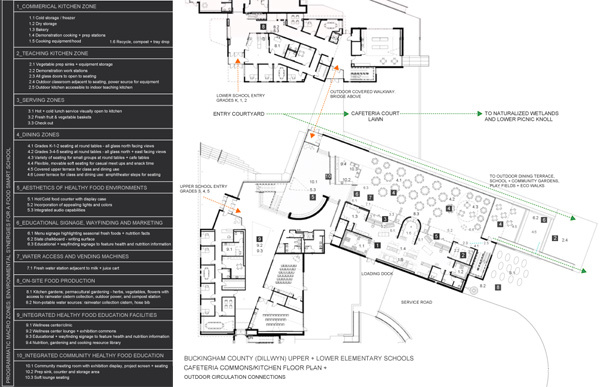
Floor plan for the cafeteria and kitchen for upper and lower elementary schools, Dillwyn, Virginia.

**Figure 3:**
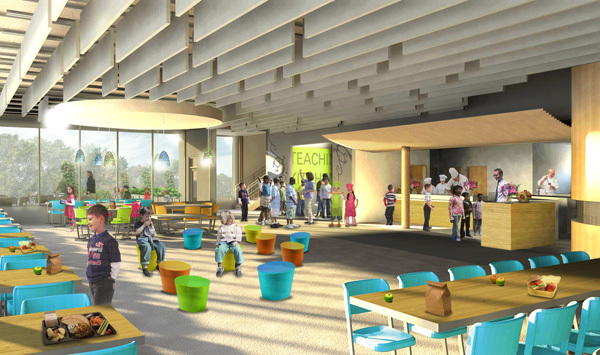
Artist rendering of open kitchen and co-located teaching kitchen for upper and lower elementary schools, Dillwyn, Virginia.

The redesigned schools incorporate a range of strategies and components, including space for school gardens and outdoor eating (Figure 1). The layout of the commercial kitchen, teaching kitchen, and serving and dining areas (Figure 2) incorporates several strategies. The open commercial and teaching kitchens are co-located (Figure 3).

The collaborative research and design process relied on 5 elements:

1. A unified vision and common goals and values. The team discussed and agreed on a commitment to the social causes of environmental sustainability, health, and education; we also shared a belief in the value of innovation and the harmonization of public and private interests.
2. Identification of required skills and resources. We identified the multidisciplinary skills required for the project, including expertise in pediatrics, obesity prevention, environmental health, multiple design dimensions, and architectural processes. The sharing of resources (eg, literature, materials, hardware, software, time) was essential.
3. A focus on connecting conceptual and practical considerations. Design strategies needed to adhere to public health principles, evidence, or best practices yet remain architecturally practical.
4. Development of a common lexicon. Differences in language between design and public health in the context of school environments were resolved, and a common lexicon was developed to make design guidelines understandable for users from different backgrounds.
5. An open and iterative culture for exchange of ideas. The guidelines were shaped through multiple phases of brainstorming, researching, and conceptual testing. They were presented in various formats at several regional and national design and public health conferences, which served as a form of external review. The integration of academic research into the operations of a private practice design firm also depended on establishing formal agreement on issues such as treatment of intellectual property, client communication protocols, peer review, public relations, and time management of research tasks.

### Identifying spatial domains and healthy eating design principles

The success of any design guidelines is measured by the degree to which they provide exact, practical, spatially based recommendations for architects, interior designers, urban planners, and other design professionals. Health-oriented design tools, such as New York City’s *Active Design Guidelines* (18,19), which promote physical activity in building design, provided inspiration and a preliminary template for our guidelines. We focused on developing a design tool that 1) identifies multiple spatial zones, or domains, that could potentially affect healthy eating in the school, 2) provides exact recommendations for the physical design of each domain, including recommendations for such details as equipment provision, adjacent spaces, and visual connections, and 3) includes testable elements for the purposes of certification and post-occupancy evaluation.

We identified 10 spatial domains: 1) commercial kitchen zone; 2) teaching kitchen zone; 3) serving zones; 4) dining zones; 5) aesthetics of health food environments; 6) educational signage, way finding, and marketing; 7) water access and vending machines; 8) on-site food production; 9) integrated healthy food education facilities; and 10) integrated healthy food community. Within each spatial domain, the recommended design strategies address 5 core principles for healthy eating design:

1. Provision of equipment and spaces that facilitate the incorporation of fresh and healthy food choices into the school and its community.
2. Provision of facilities to directly engage the school community in food production and preparation.
3. Application of evidence- and theory-based behavioral science principles to “nudge” the school community toward healthy eating behaviors and attitudes.
4. Use of building and landscape features to promote awareness of healthy and sustainable food practices.
5. Conception and articulation of school spaces as community assets to multiply the benefits of school-based healthy food initiatives.

These principles are based on the research and recommendations of several organizations, including the US Department of Agriculture (20), the Centers for Disease Control and Prevention (21), the Institute of Medicine (1), and academic research groups such as the Cornell Food and Brand Laboratory (22). The principles included in this pilot version of the guidelines include testable hypotheses rather than fully established recommendations; the guidelines will evolve as evaluation data are generated.

### Focus on synergistic design

The design of complex building projects, such as an elementary school, balances a number of synergistic goals, including environmental sustainability, safety, educational attainment, and community development. Incorporation of a new goal — healthy eating — must fit within the context of these other design goals.

In our project, for example, the open floor plan of the kitchen and cafeteria promotes engagement between food service staff and students, thereby increasing the potential for educational opportunities about fresh food sourcing and preparation (Figure 3). At the same time, the physical transparency of the kitchen facilitates passive supervision of food processing and waste management by kitchen staff and the community, thereby increasing both actual and perceived safety of foods and raising awareness of the role of waste in environmental sustainability. Higher visibility for kitchen processes also helps highlight the connection between seasonally fresh foods from local farms and school gardens and the local economy, particularly in districts like Buckingham County, Virginia, that have a strong agricultural tradition.

**Figure 4:**
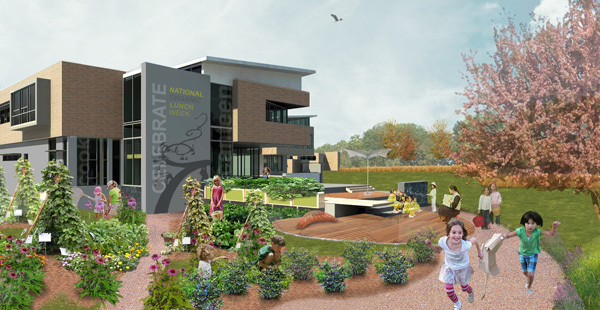
Integrated school garden and outdoor eating spaces for upper and lower elementary schools, Dillwyn, Virginia.

School architects are increasing their focus on using the building as a teaching tool for developing such perspectives as eco-literacy (23). The Healthy Eating Design Guidelines seek to use the school kitchen, cafeteria, and landscape (Figure 4) similarly to improve the health literacy of children. For example, the co-location of teaching and commercial kitchens equips the cafeteria for educational programming on initiatives such as student- and teacher-produced fresh foods. The kitchens facilitate community outreach by providing a well-equipped and inviting environment for hosting off-hour community education on nutrition, cooking skills, and other food-related topics.

The guidelines also emphasize programmatic connectivity between indoor and outdoor eating and food preparation spaces to promote flexibility in using spaces for various functions, including educational, dining, and physical activity purposes (eg, small group fitness classes such as yoga). The continuity of indoor and outdoor areas further breaks down functional boundaries.

## Applications of Design Guidelines to Architectural Design in Practice

### Architectural design process

The development and application of design tools such as the Healthy Eating Design Guidelines to produce effective yet practical building projects requires clear understanding of the design process. The architectural design process for a large-scale school project includes a series of progressive phases and a dynamic, iterative process of developing, analyzing, and testing spatial ideas and concepts. Process elements, styles, and priorities vary from project to project. However, these 4 phases are common to most building projects: programming, schematic design, design development, and construction documentation. Each phase includes its own activities and outcomes, although some phases may blend, and the overall design process is seldom linear.

The first phase, programming, focuses on 5 activities: 1) establishing goals, 2) collecting and analyzing facts, 3) formulating and testing concepts, 4) determining needs, and 5) articulating the project’s design problems (24). Programming is a critical phase for introducing healthy design considerations because the goals established during this phase largely define the purpose, priorities, and scope of the project; these factors are difficult to change later. For the second and third activities, architects review the project site, precedent studies (ie, past successful architectural projects that have relevant programming components and design ideas), and building codes, and, ideally, investigate the characteristics and cultures of the people engaged in the project. In the fourth activity, the following needs are identified: the types and functions of spaces, anticipated usage and traffic, zoning and flow, adjacencies (ie, patterns and relationships of spaces needed for functional and efficiency purposes), space standards, and anthropometrics; these are balanced with aesthetic qualities of natural daylight, materials, and construction details. Finally, the last activity involves identifying problems such as community, client, and budgetary goals and constraints and anticipated review processes. 

For our project, parameters of the Healthy Eating Design Guidelines were introduced during the programming phase. The guidelines thus became part of the architectural problem to be solved through the design process. The design team was then able to spatialize (ie, translate 2-dimensional information into 3-dimensional models to determine appropriate scales and proportions), test, and analyze the guidelines in subsequent phases. An integrated programmatic architectural analysis (eg, diagrams, requirements) results from the 5 activities of the programmatic phase.

In the second phase, schematic design, the design team begins to formulate potential design solutions, taking into account programming needs and problems. For new structures or buildings, designers often use a massing exercise to determine size (in volume) and organization of the required programmatic elements. After sizing and sequencing, the building form begins to take shape. A schematic design is often driven by a key idea or concept solution. Although details at this stage are few, schematic design plays an essential role in setting parameters for further development.

For our project, we used the guidelines during schematic design to consider the school’s role at the micro and macro levels. At the micro level, we focused on the overall layout of the cafeteria spaces (ie, cooking, eating, serving spaces). At the macro level, we considered how spaces fit in the larger context of the school learning environment and circulation paths (eg, layout of spaces such as a community meeting hall, teaching kitchen, and community gardens in relationship to classrooms and cafeteria space).

The next phase, design development, is more detailed; designers refine an overall structure by developing and integrating each key space within it. For our project, we developed floor and furniture plans for the main areas, including the layout of cafeteria serving lines.

Finally, the construction documentation phase translates decisions into a communication format for construction. Drawings are developed for all aspects of the building and adjacent landscaping. If changes are necessary to accommodate construction or code requirements, the main parameters of the program must still be maintained. All details down to the last doorknob are included in the construction documents. For our project, specifications for materials, color, and furniture in the cafeteria and other learning spaces were based on careful review of design research to promote an optimal environment for healthy eating choices and education. Once a bid is accepted and construction begins, the construction administration process ensures that the new structure conforms to the contract documents.

### Development of supporting tools and activities to accompany physical design

The physical structure of the school and landscape can be thought of as the “hardware” of the design-based health-promotion intervention. The materials and activities that are needed to help the school staff and community make optimal use of the physical structure and space post-occupancy can be thought of as supporting “software.” Such software can be delivered in various ways. The practice of providing “owner’s manuals” to school clients is growing. These manuals explain the operation of advanced systems (eg, heating, ventilation, and air-conditioning) or provide guidance on how to change grounds-keeping practices to accommodate sustainable landscaping. Similar resources and capacity for technical assistance will be needed to help schools fully use healthy design features.

Providing ideas to schools for activities that incorporate healthy design features can also be a useful form of post-occupancy support. Such activities include helping to connect the school to local farms or agricultural extension services to improve school food purchases, assisting in the operation of school gardens, or installing farmers’ markets on school grounds. Combining the physical redesign of a school (ie, design hardware) and realignment of school programs and curricula (ie, design software) shows great promise in improving the health of schoolchildren (11).

## Research Opportunities

The introduction of systematic tools such as the Healthy Eating Design Guidelines presents unique opportunities to grow the evidence base for health-promoting architecture. Research using case studies, case series, and quasi-experimental and experimental designs can shed light on the efficacy and effectiveness of proposed design strategies for improving food-related programming, curricula, purchases and consumption, and knowledge, attitudes, and norms.

Our study team is currently conducting quasi-experimental, mixed-methods evaluation of the 2 Buckingham County schools involved in our study, which opened in fall 2012. We are studying the effect of the new architecture on school-level practices and curricula; food procurement; staff attitudes and student food purchases, knowledge, attitudes, and norms. Such research has the potential to provide practice-based evidence that will inform the continued development and use of the guidelines as well as school building policies and practices in general.

## Conclusion

The Healthy Eating Design Guidelines for School Architecture is an innovation in both architecture and public health. This work represents a rare collaboration between architects and public health scientists to find new solutions for improving the school environment for children’s health. The introduction of these guidelines follows a recent trend to advance environmental design in communities. Already, New York City’s *Active Design Guidelines* (www.nyc.gov/adg) is driving the design requirements for city-owned facilities. We hope that our healthy eating–focused design guidelines for school architecture will continue to spur new research to document the effect of the health-promoting design features. New evidence will allow the design guidelines to evolve and become increasingly refined. Such undertakings promise to lead to the convergence of evidence-based architecture and evidence-based public health, providing new strategies as part of a comprehensive approach to address childhood obesity.
